# The triglyceride-to-HDL cholesterol ratio is associated with lower BMD in older adults at high altitudes: a retrospective retrospective cross sectional study

**DOI:** 10.3389/fendo.2026.1809643

**Published:** 2026-04-07

**Authors:** Chun Feng, Yulian Liu, Guilan Sheng, Haifang Shang

**Affiliations:** 1 Department of Endocrine Metabolism, Qinghai Red Cross Hospital, Xining, Qinghai, China; 2 Department of Infectious Diseases, Qinghai Red Cross Hospital, Xining, Qinghai, China

**Keywords:** bone mineral density, high altitude, insulin resistance, older adults, TG/HDL-C

## Abstract

**Purpose:**

Focusing on older adults living in high-elevation areas, this study aimed to examine the association between the TG/HDL-C ratio and the odds of low bone mineral density (BMD), providing evidence to support bone health management in this special environment.

**Methods:**

In this retrospective analysis conducted at our Hospital, we recruited 650 older adults residing in high-elevation regions between December 2022 and December 2024. Baseline characteristics were gathered using a combination of questionnaires, physical assessments, and laboratory tests. Bone mineral density (BMD) at the lumbar spine (L1–L4) and femoral neck was evaluated via dual-energy X-ray absorptiometry (DXA). Subjects were stratified into quartiles based on their TG/HDL-C ratios. To assess the link between these ratios and the likelihood of low BMD, we employed multivariate logistic regression, while restricted cubic spline (RCS) modeling and stratified analyses were utilized to examine dose-response patterns and ensure result stability.

**Results:**

As the TG/HDL-C ratio increased, participants tended to have less favorable metabolic profiles, accompanied by lower lumbar spine and femoral neck BMD values (P<0.001). The prevalence of low BMD increased from 35.8% in Q1 to 61.3% in Q4 (P<0.001). After adjusting for potential confounders (age, sex, BMI, lifestyle factors, hypertension, diabetes, and hemoglobin), a higher TG/HDL-C ratio was independently associated with higher odds of low BMD. Compared with Q1, Q4 showed higher odds of low BMD in the lumbar spine (OR = 2.86, 95%CI: 1.72–4.75; P for trend=0.003) and femoral neck (OR = 3.55, 95%CI: 1.65–7.62; P for trend=0.005). Restricted cubic spline analyses suggested a significant non-linear association between the TG/HDL-C ratio and the odds of low BMD at both sites (non-linear P = 0.021 for lumbar spine; 0.045 for femoral neck), with steeper increases in odds when the ratio exceeded approximately 1.5 and 2.0, respectively. Subgroup analyses showed consistent associations across age, sex, BMI, and comorbidity strata.

**Conclusion:**

Among older adults living at high altitudes, a higher TG/HDL-C ratio was associated with lower BMD in the lumbar spine and femoral neck, with a non-linear pattern. The TG/HDL-C ratio may serve as a practical marker to help identify individuals with a higher probability of low BMD in this setting.

## 1 Introduction

Osteoporosis (OP) resulting fragility fractures significantly increase the disability and mortality rates of the elderly population and have become a major public health challenge worldwide (1, 2). The pathophysiological mechanisms of OP have been extensively studied in plains areas, but in special geographical environments, especially high-altitude hypoxic environments, there are still many unknowns about the bone health status of the elderly population and its influencing factors (3). Epidemiological evidence suggests that environmental factors have a bidirectional regulatory effect on bone metabolism. Strong ultraviolet radiation and high physical activity in high-altitude areas may have a protective effect on bones, but chronic hypoxia, cold stress, and differences in nutritional structure may accelerate bone loss (1, 4, 5). In particular, cohort studies targeting the elderly population have found that the prevalence of osteoporosis shows a significant upward trend with increasing altitude, suggesting that the decreased adaptability of the elderly to hypoxic environments may become a potential threat to bone health (1).

In recent years, the association between metabolic syndrome and its components and osteoporosis has attracted great interest in the academic community. Dyslipidemia is considered to be the common pathological basis connecting cardiovascular disease and osteoporosis. Previous studies have explored the relationship between lipid indicators such as HDL-C and TG and bone mineral density (BMD), but the results show significant heterogeneity and even contradiction (6). HDL-C is generally considered to have anti-inflammatory protective effects, but it has been found to be negatively correlated with BMD in many elderly studies (6–8). This inconsistency may be due to the fact that single lipid indicators are greatly affected by biological variations and drug interference, and cannot fully reflect the complex insulin resistance and lipoprotein particle characteristics of the body.

TG/HDL-C, as a novel composite lipid index, integrates information from both TG and HDL-C and has been confirmed by multiple studies as a reliable alternative biomarker for insulin resistance. The TG/HDL-C ratio reflects both the triglyceride load contributing to atherosclerosis and the protective effect of HDL-C, thus providing a more comprehensive picture of lipid metabolism imbalance. It does not directly include fasting blood glucose, and fasting blood glucose levels in the elderly can fluctuate significantly due to factors such as blood glucose control and medication use. Furthermore, it is part of routine lipid testing; therefore, the TG/HDL-C ratio is readily available and cost-effective, especially in high-altitude areas with limited medical resources (9–11). Basic research has shown that insulin is a key osteogenic regulatory hormone, and insulin resistance can disrupt bone remodeling balance by damaging the insulin signaling pathway on the surface of osteoblasts, inducing oxidative stress and chronic inflammation, thereby leading to a decrease in BMD (12). In studies in plains areas, a higher TG/HDL-C ratio has been shown to be significantly associated with low BMD in postmenopausal women and patients with metabolic syndrome (13).

At high altitudes, chronic hypoxia itself can induce oxidative stress through the HIF-1α pathway and inhibit the phosphorylation of insulin receptor substrates, thereby triggering or exacerbating insulin resistance (14). This environmentally induced metabolic stress may further modify or amplify the damaging effects of lipid metabolism disorders on bone (12, 14). To our knowledge, evidence regarding the association between the TG/HDL-C ratio and BMD in high-altitude populations remains limited.

Therefore, this retrospective cross-sectional study focused on elderly populations in high-altitude areas, systematically analyzing the association between the TG/HDL-C ratio and bone mineral density (BMD). We hypothesized that a higher TG/HDL-C ratio would be associated with higher odds of low bone mineral density in older adults living at high altitudes. Elucidating this relationship will help reveal the potential mechanisms by which environmental and metabolic factors interact to influence bone health, and will also provide a simple and cost-effective clinical basis for early screening and intervention in high-risk groups for osteoporosis in high-altitude areas.

## 2 Materials and methods

### 2.1 Study population and design

Participants were selected from permanent residents who underwent health checkups and BMD screening at our hospital between December 2022 and December 2024. Inclusion criteria were as follows: (1) age ≥ 60 years; (2) long-term residence in high-altitude areas, altitude ≥ 2500 meters; (3) long-term residence in the local area for ≥ 5 years to ensure that the body has established a stable high-altitude hypoxia acclimatization state; (4) possession of complete baseline demographic information, biochemical test data and DXA BMD report. Exclusion criteria included: (1) having secondary diseases known to affect bone metabolism, such as hyperparathyroidism, chronic renal insufficiency, malignant tumors, and rheumatoid arthritis; (2) having taken anti-osteoporosis drugs, glucocorticoids, or other drugs affecting bone metabolism within the past 12 months; (3) having unreliable BMD measurements due to severe spinal deformities or fractures.

This retrospective cross-sectional study used a logistic regression model to analyze the association between TG/HDL-C and BMD, and included 16 covariates to control for confounding factors. Sample size estimation was based on the regression effect size f². Under the conditions of a two-tailed significance level of α=0.05 and a power of 1−β=0.80, the minimum required sample size, calculated according to the small effect size standard f²=0.012, was 410 cases. The formula is as follows:


$$n=\frac{{\left(Z_{1-\alpha /2}+Z_{1-\beta /2}\right)}^{2}}{f^{2}}+k+1$$

Where k represents the total number of independent variables in the model. This study ultimately included 650 participants, significantly exceeding the minimum sample size required for the analysis, indicating that the study has sufficient statistical power. Given the retrospective nature of the study and the de-identification of the data, the committee approved an exemption from obtaining informed consent from the participants.

### 2.2 Data collection and clinical assessment

Baseline information for all participants was collected by trained medical personnel using standardized questionnaires and an electronic medical record system. Demographic characteristics collected included age and sex; lifestyle factors included smoking status and alcohol consumption. Past medical history primarily recorded the prevalence of hypertension and type 2 diabetes, with diagnostic criteria based on relevant international guidelines or current medication treatment. Anthropometric indicators include Body Mass Index (BMI).

All biochemical indicators used to calculate the triglyceride/high-density lipoprotein cholesterol ratio and DXA-based BMD assessment were completed in the same health checkup and retrospectively extracted from the same time period from the electronic medical record system.

### 2.3 Biochemical indicator detection

Subjects were required to fast for at least 8–12 hours, and venous blood samples were collected the following morning. All samples were sent to the central laboratory for analysis within 2 hours of collection. The following indicators were measured using a fully automated biochemical analyzer (Hitachi 7180, Japan): TC, HDL-C, FPG, UA, Cr, and Hb.

### 2.4 BMD measurement and result definition

BMD (g/cm2) of the lumbar spine (L1-L4) and femoral neck was measured using a DXA (AKDX-09W-II, Shenzhen Aikerui Electric Co., Ltd.). Quality control calibration was performed using a phantom before daily operation, with the coefficient of variation (CV) controlled below 1.0%. According to the World Health Organization (WHO) standards, the T-score was used to assess skeletal health: a T-score ≥ -1.0 defined as normal bone mass; -2.5< T-score< -1.0 defined as low bone mass; and a T-score ≤ -2.5 defined as osteoporosis (OP). In this study, the primary endpoint was defined as low BMD. Throughout the study, all bone mineral density measurements were performed using the same DXA scanner.

### 2.5 Exposure variable

Exposure Variable: TG/HDL-C ratio. Treatment: In the statistical analysis, based on the sample distribution, the TG/HDL-C ratio was transformed into a categorical variable (Q1, Q2, Q3, Q4) according to the quartiles, where Q1 (lowest quartile) was set as reference group 1.

### 2.6 Statistical methods

R software (version 4.2.0) was used for data management and statistical analysis. Tests for homogeneity of variance and normality were performed on continuous variables. Mean ± SD was used to express normally distributed continuous data, and one-way ANOVA was used to compare groups. The Kruskal-Wallis H test was utilized to compare non-normally distributed data, which were expressed as median (interquartile range). Frequencies (percentages) were used to characterize categorical variables, and the chi-square test was used to evaluate group differences.

A multivariate logistic regression model was used to assess the association between the TG/HDL-C ratio and the risk of low BMD, with results expressed as odds ratios (OR) and 95% confidence intervals (95% CI). A linear trend test was used to assess the statistical significance of the dose-response relationship.

Given the often non-linear relationship between metabolic indicators and disease risk, this study further employed a restricted cubic spline (RCS) model to simulate the association between the TG/HDL-C ratio and the risk of low BMD. The RCS model included three nodes located at the 10%, 50%, and 90% quantiles of the distribution. The significance of the non-linear term was assessed using the Wald test; a p-value< 0.05 indicated a non-linear relationship, and a smooth curve was plotted to visually represent the inflection point of risk change. All statistical tests were two-tailed, and a p-value< 0.05 was considered statistically significant.

## 3 Results

### 3.1 Baseline data

This retrospective cross sectional study included 650 older adults living in high-altitude areas (**Table 1**). The mean age of the total population was (68.7 ± 6.4) years, and 364 participants were female. As expected, participants with higher TG/HDL-C levels in Q4 had significantly higher TG levels and significantly lower HDL-C levels (P< 0.001 for both). The proportion of women decreased significantly across all quartiles, from 63.0% in Q1 to 48.5% in Q4 (P = 0.035), while there were no significant differences in age among the groups. Compared to participants in Q1, participants in Q4 exhibited more unfavorable metabolic characteristics. Specifically, BMI steadily increased across the TG/HDL-C quartiles (P<0.001). With increasing TG/HDL-C ratios, the prevalence of comorbidities such as hypertension and type 2 diabetes significantly increased (P = 0.003 and P<0.001, respectively). Consistent with these findings, FPG and uric acid levels were also significantly elevated in the higher quartiles (both P<0.001). Notably, hemoglobin levels were generally elevated in this high-altitude population, showing a significant upward trend across all quartiles (P<0.001). The proportion of current smokers was higher in the higher quartiles (P = 0.042), while there were no significant differences in alcohol consumption, TC, LDL-C, and creatinine levels among the groups. Regarding bone health indicators, there was a significant inverse relationship between the TG/HDL-C ratio and bone mineral density (BMD).

**Table 1:** Baseline data.

Variables	Total number of cases (n=650)	Q1(<0.90)(n=162)	Q2(0.90-1.45) (n=163)	Q3(1.45-2.20) (n=162)	Q4(≥2.20) (n=163)	P
Age,years	68.7 ± 6.4	69.2 ± 6.8	68.8 ± 6.3	68.5 ± 6.1	68.3 ± 6.5	0.582
Female	364(56.0%)	102(63.0%)	95(58.3%)	88(54.3%)	79(48.5%)	0.035
BMI(kg/m^2^)	24.6 ± 3.4	22.8 ± 3.0	24.1 ± 3.2	25.3 ± 3.5	26.2 ± 3.8	<0.001
Current Smoker	132(20.3%)	24(14.8%)	30(18.4%)	35(21.6%)	43(26.4%)	0.042
Alcohol Drinking	108(16.6%)	19(11.7%)	25(15.3%)	29(17.9%)	35(21.5%)	0.088
Comorbidities						
Hypertension	328(50.5%)	65(40.1%)	78(47.9%)	88(54.3%)	97(59.5%)	0.003
T2DM	138(21.2%)	18(11.1%)	28(17.2%)	39(24.1%)	53(32.5%)	<0.001
TG(mmol/L)	1.85(1.15-2.65)	0.95(0.82-1.08)	1.42(1.25-1.62)	1.98(1.75-2.15)	2.95(2.45-3.80)	<0.001
HDL-C(mmol/L)	1.22 ± 0.31	1.48 ± 0.28	1.29 ± 0.24	1.15 ± 0.20	0.98 ± 0.18	<0.001
TG/HDL-C	1.58(0.95-2.40)	0.68(0.55-0.82)	1.15(0.98-1.32)	1.75(1.55-2.05)	3.10(2.55-4.20)	<0.001
TC(mmol/L)	4.92 ± 1.05	4.75 ± 0.98	4.88 ± 1.02	4.98 ± 1.06	5.08 ± 1.12	0.065
LDL-C(mmol/L)	2.85 ± 0.82	2.78 ± 0.78	2.82 ± 0.80	2.88 ± 0.84	2.92 ± 0.86	0.324
FPG(mmol/L)	5.65 ± 1.45	5.25 ± 1.10	5.48 ± 1.25	5.75 ± 1.52	6.12 ± 1.85	<0.001
Uric Acid(μmol/L)	358.5 ± 92.4	325.2 ± 82.5	348.6 ± 88.3	368.4 ± 94.1	392.5 ± 98.6	<0.001
Hemoglobin(g/L)	156.4 ± 16.2	148.5 ± 15.1	154.2 ± 15.8	158.8 ± 16.5	164.2 ± 17.1	<0.001
Creatinine(μmol/L)	76.5 ± 18.2	74.2 ± 16.5	75.8 ± 17.1	77.4 ± 18.8	78.6 ± 19.5	0.152
Lumbar spine L1-L4)BMD(g/cm^2^)	0.905 ± 0.152	0.958 ± 0.145	0.925 ± 0.148	0.886 ± 0.154	0.852 ± 0.158	<0.001
Femoral neck BMD(g/cm^2^)	0.745 ± 0.125	0.788 ± 0.118	0.762 ± 0.122	0.735 ± 0.126	0.695 ± 0.132	<0.001
Low bone mass/OP	315(48.5%)	58(35.8%)	72(44.2%)	85(52.5%)	100(61.3%)	<0.001

### 3.2 Correlation between TG/HDL-C ratio quartiles and BMD

In multivariate regression analysis, the results showed a significant positive correlation between elevated TG/HDL-C ratio and the risk of low BMD. Taking the L1–4 lumbar spine as an example, in Model 1 (unadjusted or slightly adjusted), the 95% CIs of Q2, Q3, and Q4 compared to Q1 were 1.45 (0.92-2.28), 2.56 (1.64-3.99), and 4.85 (3.05-7.72), respectively, with a significant overall trend (P for trend< 0.001). In Models 2 and 3, which adjusted for demographic characteristics, lifestyle factors, and metabolic covariates, this positive association remained robust; in Model 3, the odds ratios of Q3 and Q4 were 1.95 (1.18-3.22) and 2.86 (1.72-4.75), respectively, and the trend test was also statistically significant (P for trend = 0.003), suggesting an independent association between high TG/HDL-C levels and low lumbar BMD (**Table 2**).

**Table 2:** Association between quartiles of the TG/HDL-C ratio and odds of low BMD based on multivariable logistic regression analysis.

Model	Q1	Q2	Q3	Q4	P for trend
Lumbar spine L_1-4_					
Model 1	Ref	1.45(0.92-2.28)	2.56(1.64-3.99)	4.85(3.05-7.72)	<0.001
Model 2	Ref	1.38(0.86-2.21)	2.35(1.48-3.72)	4.10(2.52-6.68)	<0.001
Model 3	Ref	1.32(0.81-2.15)	1.95(1.18-3.22)	2.86(1.72-4.75)	0.003
Femoral neck					
Model 1	Ref	1.55(0.72-3.35)	3.05(1.52-6.12)	6.25(3.15-12.4)	<0.001
Model 2	Ref	1.42(0.65-3.12)	2.75(1.35-5.62)	5.45(2.68-11.1)	<0.001
Model 3	Ref	1.35(0.60-3.05)	2.25(1.05-4.82)	3.55(1.65-7.62)	0.005

The analysis results for the femoral neck were consistent with those for the lumbar spine, and the intensity was similar or higher. In Model 1, the odds ratios for Q2 to Q4 were 1.55 (0.72-3.35), 3.05 (1.52-6.12), and 6.25 (3.15-12.4), respectively, with P for trend< 0.001. After full adjustment (Model 3), the odds ratios for Q3 and Q4 were 2.25 (1.05-4.82) and 3.55 (1.65-7.62), respectively, and the trend remained significant (P for trend = 0.005). Overall, a higher TG/HDL-C ratio significantly increased the risk of low BMD in the lumbar spine and femoral neck, and this association remained robust after multivariate adjustment.

### 3.3 Exploring nonlinear dose-response relationships using RCS

The results showed a significant overall association between TG/HDL-C and lower BMD risk in both the L1-L4 lumbar spine and the femoral neck region (all P< 0.001), exhibiting a non-linear trend. For the L1-L4 lumbar spine, the curve showed a slight decreasing trend at lower TG/HDL-C levels, followed by a rapid increase, with statistical significance for non-linearity (P = 0.021). When the TG/HDL-C ratio exceeded approximately 1.5, the risk of reduced BMD increased significantly with increasing ratio, and the rate of risk increase gradually accelerated.

**Figure 1:**
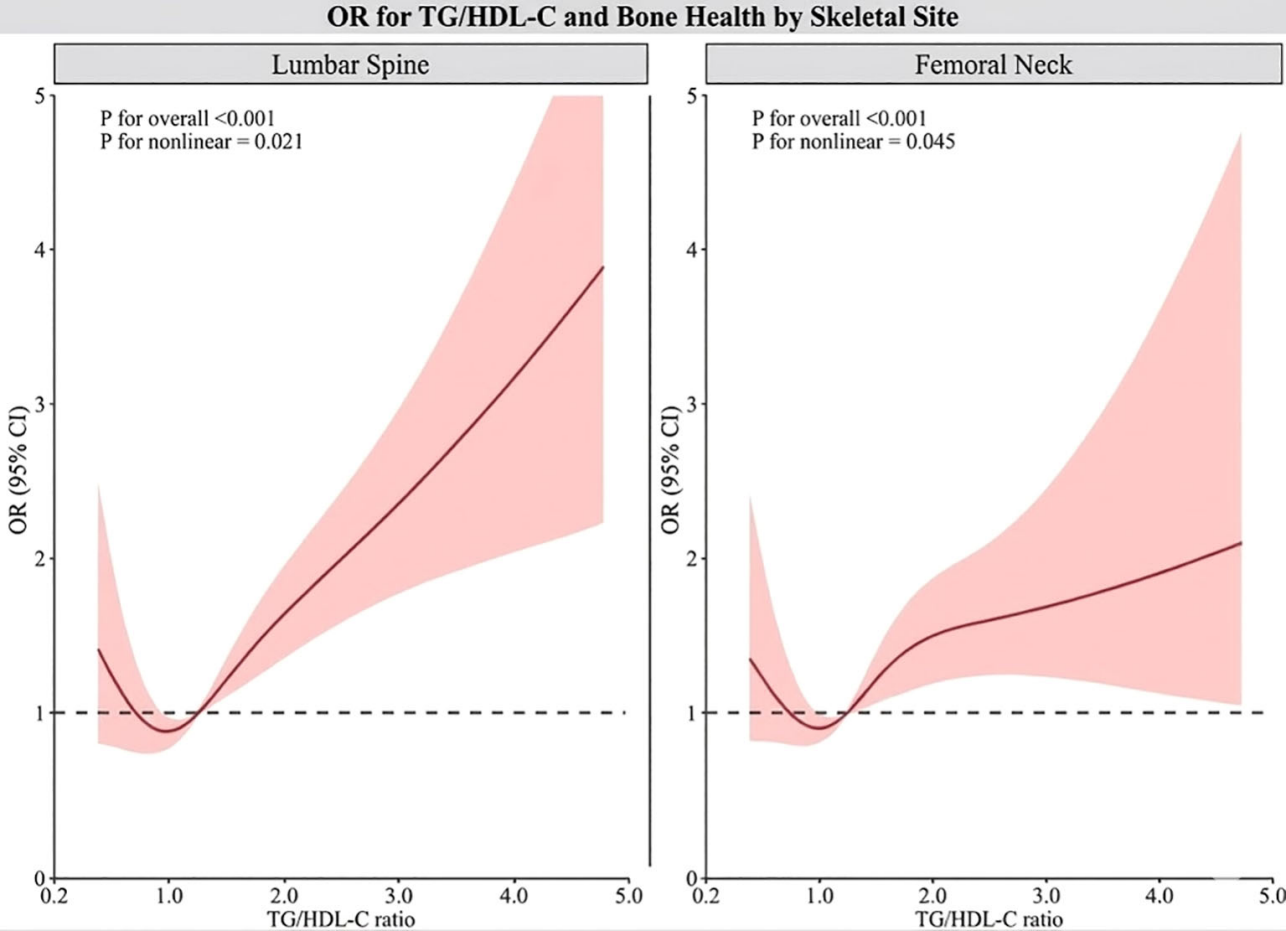
RCS explores the nonlinear dose-response relationship between TG/HDL-C ratio and BMD. The model was adjusted in the same way as Model 3, including age, sex, BMI, smoking status, alcohol consumption, hypertension, type 2 diabetes, fasting blood glucose, uric acid, hemoglobin, total cholesterol, LDL-C, and creatinine. The solid line represents the adjusted odds ratio, and the shaded area represents the 95% confidence interval.

In the femoral neck region, the dose-response curve also exhibited a pattern of initial flattening followed by an increase. Nonlinear trend testing suggested that the association was not simply linear (P = 0.045). When the TG/HDL-C ratio was low, the risk of BMD did not change significantly, but after the ratio exceeded approximately 2.0, the risk began to gradually increase, showing a more pronounced increase at higher levels. Overall, RCS analysis indicated that the relationship between TG/HDL-C and BMD was not a constant linear one; higher TG/HDL-C levels corresponded to a higher risk of decreased BMD, while changes at low to moderate levels were relatively gradual (**Figure 1**).

### 3.4 Subgroup analysis

In further subgroup analyses, the association between the TG/HDL-C ratio and the lower risk of BMD in the lumbar L1–4 and femoral neck remained consistent across multiple baseline characteristics. Taken together, the inverse correlation between elevated TG/HDL-C and BMD persisted across the majority of stratified analyses. The uniformity of this trend underscores the robustness of the observed association.

For L1–4 lumbar spine bone density (BMD), in the age subgroups, the odds ratio (OR) was 1.75 (95% CI: 1.05-2.92) in individuals<70 years of age, rising to 2.40 (95% CI: 1.45-3.98) in individuals ≥70 years of age. The interaction between the two groups was not statistically significant (P = 0.21), indicating that age did not significantly affect the relationship between TG/HDL-C and lumbar spine BMD. In the sex subgroups, both males (OR = 2.10, 95% CI: 1.20-3.65) and females (OR = 1.80, 95% CI: 1.15-2.82) showed significantly increased risk, with an interaction P = 0.27. Similarly, no change in the direction of the effect was observed between sexes. The results for subgroups also showed similar trends: although there were some differences in the absolute values of ORs among the subgroups, high TG/HDL-C levels were consistently associated with a higher risk of low lumbar spine BMD, and the interaction P values among the subgroups were all >0.05, suggesting that the above factors did not significantly modify the main effect.

In subgroup analyses of femoral neck BMD, the results also showed a consistent positive association between TG/HDL-C and reduced BMD risk across different population characteristics. Regardless of whether the subgroups were young or old, male or female, different BMI levels, smoking or non-smoking, drinking or non-drinking, or had hypertension or type 2 diabetes, a high TG/HDL-C ratio was associated with an increased risk of low femoral neck BMD. The odds ratios (ORs) for most subgroups ranged from 1.7 to 3.5, with variations between subgroups primarily reflecting effect size rather than effect direction. All interaction P-values were not statistically significant (all P > 0.05), further indicating that different baseline characteristics had a relatively small impact on this association, and no significant effect modification was observed.

Overall, subgroup analyses of both the lumbar L1–4 and femoral neck did not show that the relationship between TG/HDL-C and BMD was significantly affected by age, sex, BMI, smoking, alcohol consumption, hypertension, and type 2 diabetes. This suggests that the TG/HDL-C ratio is a relatively stable indicator of the risk of decreased BMD in elderly people at high altitudes, and its adverse effects remain consistent across various clinical contexts (**Table 3**).

**Table 3:** Subgroup analysis of TG/HDL-C and low BMD risk in lumbar L1–4 and femoral neck.

Baseline data		n	Lumbar spine L1-4	P	Femoral neck	P
			OR(95%CI)		OR(95%CI)	
Age	<70 years	292	1.75(1.05-2.92)		2.05(1.20-3.50)	0.24
	≥70 years	358	2.40(1.45-3.98)		3.00(1.70-5.30)	
Gender	Male	286	2.10(1.20-3.65)	0.27	2.55(1.45-4.60)	0.31
	Female	364	1.80(1.15-2.82)		2.10(1.30-3.40)	
BMI	<24 kg/m²	260	2.35(1.30-4.25)	0.11	3.05(1.65-5.70)	0.10
	≥24 kg/m²	390	1.65(1.00-2.72)		1.95(1.10-3.10)	
Smoking status	Current or former smokers	132	2.30(1.20-4.40)	0.29	2.95(1.55-5.60)	0.29
	Never smokes	518	1.90(1.15-3.15)		2.20(1.30-3.65)	
Drinking status	Currently drinking alcohol	108	2.15(1.10-4.20)	0.38	2.75(1.35-5.60)	0.36
	Not drinking alcohol	542	1.85(1.15-2.95)		2.10(1.30-3.40)	
hypertension	Yes	328	2.60(1.45-4.68)	0.14	3.25(1.70-6.20)	0.12
	No	322	1.70(1.02-2.84)		1.95(1.10-3.45)	
Type 2 diabetes	Yes	138	2.90(1.50-5.60)	0.17	3.50(1.75-7.00)	0.18
	No	512	1.95(1.20-3.15)		2.15(1.30-3.55)	

## 4 Discussion

This retrospective cross sectional study investigated the association between the TG/HDL-C ratio and BMD in an elderly population living at high altitudes. Our main findings indicate that in this specific population exposed to chronic hypoxia, an elevated TG/HDL-C ratio was significantly associated with decreased BMD in the lumbar spine and femoral neck, as well as an increased risk of osteoporosis. RCS analysis revealed a potentially non-linear dose-response relationship: the risk of low BMD increased sharply when the TG/HDL-C ratio exceeded a certain threshold. This independent association remained robust even after adjusting for age, BMI, hemoglobin levels, and various metabolic comorbidities.

Previous research findings on lipid indicators and bone health remain controversial. Some recent studies suggest that moderate lipid accumulation may have a protective effect on bones by increasing mechanical load, the so-called “obesity paradox” (15, 16). Cui et al. (15) found in a study of American adults that there was a saturation effect between lipid accumulation index and BMD, and that they were positively correlated only within a certain range. However, increasing evidence supports the “lipotoxicity” hypothesis, that is, excessive lipid accumulation will have an adverse effect on bone metabolism (17). This inconsistency may stem from the fact that a single lipid indicator cannot accurately reflect the complex state of insulin resistance in the body. In contrast, the TG/HDL-C ratio, as a comprehensive surrogate indicator of insulin resistance and atherogenic lipid profile, has better predictive value than a single lipid parameter (18, 19). The results of this study are consistent with the findings of Chen et al. (20) in postmenopausal women and echo the findings of Wen et al. (21) on TyG index (which is highly correlated with TG/HDL-C) and osteoporotic fracture risk, further suggesting the universality of this composite index in identifying bone health risks in different populations.

The association between the TG/HDL-C ratio and bone loss observed in this study may be related to insulin resistance. An elevated TG/HDL-C ratio is considered a marker of systemic insulin resistance (19). According to a recent review, the insulin signaling pathway plays an important anabolic role in osteoblast proliferation and differentiation (22). Therefore, impaired insulin sensitivity may be accompanied by reduced bone matrix synthesis and alterations in the Wnt/β-catenin signaling pathway (22, 23). Lipid metabolism disorders are often associated with abnormal proliferation of bone marrow adipose tissue. Experimental studies have shown that elevated TG levels may reflect a tendency for mesenchymal stem cells to differentiate into adipocytes, which may lead to a pro-inflammatory bone marrow microenvironment (24, 25). However, this study did not directly measure these mechanistic pathways, and therefore should be interpreted as a biologically plausible explanation rather than a proven causal mechanism.

The unique characteristics of high-altitude environments may be an important background factor in our study population. Participants were exposed to hypoxia for a long time, and the elevated hemoglobin levels indicated physiological adaptation. Sustained hypoxia exposure can activate the hypoxia-inducible factor-1α (HIF-1α) signaling pathway (26). Experimental evidence suggests that pathological hypoxia may disrupt the coupling balance between osteoblasts and osteoclasts through NF-κB-related pathways (26). In addition, Mendelian randomization studies have shown a possible association between hypoxia, dyslipidemia, and osteoporosis (27). Individuals with a high TG/HDL-C ratio are often characterized by enhanced oxidative stress, while the antioxidant function of HDL-C may be impaired under hypoxia and metabolic stress conditions (27, 28).

The clinical significance of this study lies in the fact that the TG/HDL-C ratio, as a simple calculation indicator derived from routine biochemical tests, has extremely high cost-effectiveness. For high-altitude areas with relatively scarce medical resources, this indicator can serve as an early screening tool for identifying high-risk groups for osteoporosis. The thresholds recommended by RCS analysis can be used for risk stratification and priority screening, meaning that older adults with significantly higher rates should be given more proactive attention to their bone health. Importantly, this study is observational in nature; therefore, causal inferences cannot be established. The observed associations should be interpreted as correlations rather than proof of causality. Further prospective cohort studies and mechanistic investigations are warranted to clarify the underlying biological pathways.

This study has several limitations. The retrospective cross-sectional design prevents causal inference and assessment of temporal direction; therefore, reverse causality cannot be ruled out. Despite adjusting for a range of demographic, lifestyle, and metabolic covariates, residual confounding factors may still exist, particularly those missing from the database related to bone health, such as serum 25-hydroxyvitamin D, parathyroid hormone, dietary calcium intake, objectively measured physical activity, and detailed menopausal information for women, including years since menopause and hormone therapy status. These factors are particularly important at high altitudes, as UV exposure, hypoxia, and lifestyle patterns may collectively influence mineral metabolism and bone remodeling. This single-center study, including older adults undergoing health checks, may limit the general applicability of the findings. Therefore, prospective cohort studies with repeated measures are necessary to better characterize bone-related confounding factors and employ mechanistic/causal relationship analysis methods.

## 5 Conclusion

In older adults living at high altitudes, a higher TG/HDL-C ratio was substantially linked to a lower bone mineral density (BMD). This simple metabolic indicator may help identify high-risk individuals for impaired bone health in this unique environment, suggesting that we should emphasize the potential benefits of lipid metabolism management for bone health in geriatric healthcare at high altitudes.

## Data Availability

The original contributions presented in the study are included in the article/supplementary material. Further inquiries can be directed to the corresponding author.
